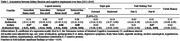# Association between kidney dysfunction and cognitive impairment in community‐dwelling older adults in Taiwan

**DOI:** 10.1002/alz70860_100341

**Published:** 2025-12-23

**Authors:** Yi‐Chun Lin, Jen‐Hau Chen, Jeng‐Min Chiou, Yen‐Ching Chen

**Affiliations:** ^1^ Institute of Epidemiology and Preventive Medicine, College of Public Health, National Taiwan University, Taipei, Taiwan; ^2^ College of Medicine, National Taiwan University, Taipei, Taiwan; ^3^ National Taiwan University Hospital Yunlin Branch, Yunlin, Taiwan; ^4^ Institute of Statistics and Data Science, National Taiwan University, Taipei, Taiwan; ^5^ Institute of Statistical Science, Academia Sinica, Taipei, Taiwan; ^6^ National Taiwan University, Taipei, Taiwan

## Abstract

**Background:**

Kidney function has been shown to predict cognitive impairment in previous studies. However, limited longitudinal studies have investigated the association between kidney function and cognitive impairment. This study aimed to explore this association in a non‐demented elderly population.

**Method:**

This prospective cohort study (2011–2019) is based on the Taiwan Initiative for Geriatric Epidemiological Research (TIGER). After exclusion, 515 non‐demented older adults were included at baseline (2011‐2013). Kidney function was evaluated by calculating the estimated glomerular filtration rate (eGFR) using the Chronic Kidney Disease Epidemiology Collaboration (CKD‐EPI) equation. Kidney dysfunction was defined as participants with an eGFR <60 mL/min/1.73 m^2^ or the existence of proteinuria. Cognitive assessment includes global and domain‐specific cognition (memory, executive function, verbal fluency, and attention), using the Taiwanese version of the Montreal Cognitive Assessment and a battery of neuropsychological tests, respectively. The generalized linear mixed model was utilized to explore associations of kidney function with cognitive impairment over time adjusting for important covariates [e.g., age, sex, years of education, apolipoprotein E ε4 status, depressive symptoms, body mass index (BMI), cigarette smoking, alcohol consumption, comorbidities, and serum levels of biomarkers, including interleukin‐6 (IL‐6) and C‐reactive protein (CRP)].

**Result:**

The baseline mean age of this population was 72.7. The presence of kidney dysfunction at baseline was associated with poor performance in some cognitive domains as below. Baseline kidney dysfunction was significantly associated with poor performance of memory over time (immediate theme recall: β = ‐0.07, immediate free recall: β = ‐0.06, delayed theme recall: β = ‐0.06, delayed free recall: β = ‐0.05). Similar findings were found for executive function (Trail Making Test part B: β = ‐0.07). These associations became more evident in women or participants with higher years of education (β = ‐0.05 to ‐0.12). However, no significant association was observed for global cognition, attention, and verbal fluency.

**Conclusion:**

Baseline kidney dysfunction was associated with worse cognitive performance over time in older adults. Early screening for kidney dysfunction and cognitive impairment is essential for implementing preventive interventions to slow the progression of dementia. Further research is needed to elucidate the underlying mechanism between kidney dysfunction and cognitive impairment.